# Effect of laterality and fatigue in peroneal electromechanical delay

**DOI:** 10.1051/sicotj/2022018

**Published:** 2022-05-26

**Authors:** Dimitrios A. Flevas, Evangelos Pappas, Stavros Ristanis, Giannis Giakas, Marios Vekris, Anastasios D. Georgoulis

**Affiliations:** 1 Orthopaedic Sports Medicine Center, Department of Orthopaedic Surgery, University of Ioannina Ioannina 45110 Greece; 2 Department of Arthroscopy and Orthopaedic Surgery, Metropolitan Hospital Neo Faliro, Athens 18547 Greece; 3 School of Medicine and Illawarra Health and Medical Research Institute, The University of Wollongong Wollongong NSW 2522 Australia; 4 Department of Physical Education and Sport Science, Institute of Kinesiology, Research Centre Iason, University of Thessaly Trikala 4100 Greece; 5 Department of Orthopaedic Surgery, University of Ioannina Ioannina 45110 Greece

**Keywords:** III, Electromechanical delay, Ankle, Laterality, Ankle instability, Fatigue, Reaction time

## Abstract

*Introduction*: Extremity dominance is one of the intrinsic factors that have been identified for ankle sprains. Electromechanical delay (EMD) is an integral part of the peroneal motor response and, therefore, substantial in preventing ankle sprains. This study aimed to investigate the effect of laterality on EMD times before and after fatigue. *Methods*: Fifteen healthy male volunteers participated in the study. Measurements were taken with the ankle in a neutral (0°) position, and all subjects followed an isokinetic fatigue protocol. Repeated ANOVA was used for statistical analysis, and the *α* level was set a priori at *p* ≤ 0.05. *Results*: No significant difference was noted in EMD times between the dominant and non-dominant legs of the volunteers (*p* = 0.940). Fatigue caused a significant increase in EMD by 10–15 ms (*p* = 0.003), while the leg × fatigue interaction was not significant (*p* = 0.893). *Conclusions*: In a non-injured athlete, both ankles seem to be under the same protection of the reactive response of the peroneal muscles. Therefore, athletes should be aware that both their extremities are equally exposed to the danger of an ankle injury. Also, fatigued ankles demonstrate longer EMD times, implying that improving resistance to fatigue may add another layer of protection that has the potential to prevent ankle sprain recurrence.

## Introduction

One of the most common single types of acute sports trauma that accounts for 14% of all visits to the emergency room is the ligamentous sprain injury which is followed by a significant socioeconomic cost as well [1–4]. Among the ligamentous sprain injuries, lateral ankle sprains are most commonly encountered during athletic or occasional daily activities [5–14]. It is reported that almost 70% of the general population have incurred an ankle injury during their lifetime [15, 16]. The most common ankle injury is the one that combines rapid inversion and internal rotation of the foot with a subsequent injury to the lateral ligaments of the ankle [1, 16–18]. Although this injury is usually assumed to be benign, a group of patients will develop recurrent sprains and chronic ankle instability [9, 19, 20]. The peroneal muscles seem to play a preventive role in the lateral ankle sprain by preventing excessive inversion [3, 21–24].

Furthermore, various risk factors for ankle sprains have been described and classified. One of these classifications divides them into extrinsic and intrinsic. As extrinsic risk factors are defined as those that come from outside of the body, while intrinsic factors are those from within the body, such as height, weight, and gender [3, 25, 26]. Most proposed risk factors remain controversial [26], while different conclusions are reported [9, 20, 21, 27, 28].

One way to approach the protective action of the peroneal muscles is to assess their response by measuring their electromechanical delay (EMD) [22]. EMD is defined as the time interval from the stimulation of a muscle by the alpha motoneuron to the first detected movement this muscle produces at a given joint [29]. It has been reported that the quantification of peroneus longus EMD can be used to assess ankle instability, which has been verified to be sensitive to musculotendinous stiffness at the ankle [30–32]. EMD of the peroneal muscles can be affected by many factors, including fatigue [33, 34].

Considering that the peroneal muscles are of significant interest in preventing ankle sprains and that no consensus has been reached regarding extremity dominance as an intrinsic risk factor, this study has two purposes. It investigates the effect of (1) laterality and (2) fatigue on peroneal EMD reaction times.

## Materials and methods

### Participants

Fifteen healthy male volunteers who were all amateur athletes on a regional level participated in the study. They had a mean age of 32.3 years (mean ± SD, age: 32.3 ± 3.11) and a mean Tegner activity score of 7.06 (range: 6–8, SD: 0.57). Eleven participants declared as dominant extremity their right leg and 4 participants their left leg.

None of them had any history of surgery or fracture on either lower extremity and was not under any medication or treatment for ankle injury for at least 6 months before the study. Furthermore, none of them had any neurological problems.

### Clinical evaluation

All participants were clinically evaluated before data collection, and the same clinician evaluated the same conditions. First, the investigator explained the study protocol and obtained informed consent (on a form approved by the senior author’s institute). In order to exclude any acute injury, a physical examination was performed on each participant. Furthermore, all participants completed the lower extremity functional scale (LEFS) and the Tegner scale.

This study received ethics approval from the scientific committee of the senior author’s institute.

### Electromechanical delay

All subjects underwent torque measurements for both ankles using an isokinetic dynamometer (Biodex System 3, Biodex Corp Shirley, NY, USA). The participants sat on the testing chair with their backs at 70° inclination. The dynamometer tilt was set at 50°, and the dynamometer rotation was set at 0°. The position of the knee was at 70° of flexion and that of the ankle at 10° of plantarflexion. After positioning, the lower extremity was secured tightly with straps and also the upper body of the participant.

A wireless 8-channel EMG system (Telemyo 2400T, Noraxon, Scottsdale, Arizona, USA) was used to record the EMG data, and these data were displayed online on a computer using dedicated software (MyoResearch XP, Noraxon, Scottsdale, Arizona, USA). In order to obtain EMG from the peroneus longus (PL), circular, preamplified, pre-gelled Ag/AgCl electrodes with a 10-mm diameter and fixed inter-electrode spacing of 20 mm (Noraxon) were used bilaterally. The same examiner placed the two active electrodes bilaterally 3 cm distally to the fibular head along the course of the peroneal muscle belly. Before the electrode placement, the skin in the area was shaved, abraded, and cleaned with isopropyl alcohol. The tibial tuberosity was used for placing the reference electrode [35]. In order to avoid artefacts due to movement, the electrodes were secured with surgical tape.

Each participant completed his testing in a single session. The isokinetic and EMG equipment was calibrated and “zeroed-off” as per the manufacturers’ recommendations. The order of the dominant and non-dominant lower extremity test was randomized.

The EMG signals were acquired at a sampling rate of 1000 Hz. The root-mean-square (RMS) amplitude for each muscle burst was calculated as follows: the raw EMG signals were full-wave rectified; high-pass filtered with a Butterworth filter to remove movement artefacts with a cut-off frequency of 20 Hz. A 100-ms RMS algorithm was used to smooth the signals. Accordingly, the protocol developed by Zhou et al. was used to measure EMD using the isokinetic dynamometer and the surface EMG unit [36]. This protocol suggests that the onset of torque development is defined as a 3.6-N·m deviation above the baseline level. The onset of EMG activity is defined as a 15 μV deviation above the baseline EMG signal.

Participants were asked to perform 5 maximal isometric contractions with the ankle in neutral (0°), and measurements were taken. The first and the last contraction were not taken into account, and the EMD values of the middle three contractions were averaged.

### Fatigue protocol

An isokinetic fatigue protocol was followed after collecting the isometric data for the non-fatigued state for both ankles of each subject. During this protocol, which was followed in previous research [37], each participant executed concentric contractions for ankle eversion and inversion until eversion torque fell below 50% of initial torque for three consecutive repetitions. After achieving a fatigued state, the same isometric data collection protocol was followed.

The whole testing protocol has been used before and described in the literature [22, 35, 36]. Our previous work [22] examined the EMD time reactions in patients with chronic ankle instability under different angles and fatigue. In this work, the testing protocol is used to assess an ankle sprain factor before and after fatigue, and we are only using data from the neutral position in the current paper.

### Statistical analysis

Repeated ANOVA (analysis of variance) was used for statistical analysis to assess the effect of laterality and fatigue on EMD. The *α* level was set a priori at *p* ≤ 0.05.

## Results

The results of the study revealed no significant difference regarding laterality. The EMD times for the dominant leg of the participants had a mean value of 124 ms (23) and for the non-dominant leg, 122 ms (24). The difference between these EMD times was not significant (*p* = 0.940).

Mean EMD times after the fatigue protocol was 134 ms (24) for the dominant leg of the participants and 137 ms (38) for the non-dominant leg. Fatigue caused a significant increase in EMD by 10 and 15 ms, respectively (*p* = 0.003), while the leg × fatigue interaction was not proven significant (*p* = 0.893) (Table 1).

**Table 1:** Electromechanical delay (EMD) in msec for leg laterality and fatigue.

	Dominant		Non-dominant	
	Before fatigue	After fatigue	Before fatigue	After fatigue
EMD mean (SD)	124 (23)	134 (24)	122 (24)	137 (38)

## Discussion

Lateral ankle sprains have been reported to be the most common musculoskeletal injuries in patients considered physically active [1–16]. The anatomic area, including the foot and ankle joints, is regarded as the most common area of orthopaedic injuries [38] and can lead to recurrent sprains and chronic ankle instability [9, 19, 20]. This study aimed to assess the effect of laterality and fatigue on peroneal EMD times in healthy athletes. The findings reported here suggest that: (1) the dominant and the non-dominant extremities of an amateur athlete do not present any significant differences in peroneal EMD times, and (2) fatigued ankles demonstrate longer EMD times.

There are some limitations noted in this study. First, only male subjects were tested, which eliminates the generalizability of the findings to females. As this is laboratory research, the task that subjects were tested at was not functional; they may exhibit a different behaviour under more realistic tasks. EMD was measured during a volitional contraction that does not account for reflexive contractions that may play an important role in preventing ankle sprains. Finally, the small sample size raises the possibility of type II error.

An important component for preventing a lateral ankle sprain is the peroneal muscles [39, 40]. These muscles constitute the primary evertors of the foot and play a significant role in maintaining foot position during movement and functional activity by producing an eccentric force during inversion [17, 18, 22, 24]. The time these muscles need to react after the mechanoreceptors in the lateral ankle ligaments are activated the time they have to offer protection against a lateral ankle sprain [40–44]. Subsequently, a longer peroneal reaction time may increase the risk of a lateral ankle sprain [45] and has been proposed among the aetiologies of this entity [3, 46]. This reaction time can be assessed with the EMD, which can highlight the true effectiveness of the muscles [22, 29, 30, 32].

This work correlates the peroneal reaction time with a debatable ankle sprain risk factor. There has not been an investigation of laterality as an ankle sprain factor with the use of EMD. A classification divides risk factors for an ankle injury into extrinsic or intrinsic. Intrinsic factors for ankle injuries have been proposed a previous sprain, foot type and size, ankle instability, height, weight, generalized joint laxity, lower extremity strength, anatomic malalignment and limb dominance [26]. Although there is a partial consensus between authors regarding orthosis, foot type and generalized joint laxity, most proposed risk factors remain controversial [26]. Regarding extremity dominance, there are different conclusions reported. Ekstrand and GiIlquist [27] and Yeung et al. [9] report increased risk for the dominant ankle with a higher incidence of ankle sprain [20]. Surve et al. [21] report no differences in the incidence of ankle sprains between dominant and non-dominant ankles in soccer players [21]. Baumhauer et al. [28] found no statistically significant differences between the injured and uninjured ankles upon examining limb dominance and ankle ligament stability. However, they report an increased risk of ankle sprain in the left ankle for left low extremity dominant players [28]. The literature seems to be divided regarding laterality. Limb dominance has been reported as a possible risk factor that may increase incidence [9, 20, 26, 27, 47]. However, some studies reported no increased incidence of ankle sprains between dominant and non-dominant lower extremities [21, 48]. In the study conducted by Slevin et al. [20], only the dominant leg of the participants was assessed, based on the claim of Yeung et al. [9] of a higher incidence of ankle sprains. Subsequently, the results of this study were in favour of limb dominance being a risk factor for ankle sprains since this parameter has been accepted as a priori situation. There is also a study with reported results that are not clearly in favour or not of limb dominance. This study suggests that although no statistically significant differences between the injured and uninjured ankles were found, there is an increased risk of sprain in the left ankle for left dominant players [28]. However, these contrasting findings may result from different study designs or different methods used for data analysis [49].

Since it seems that no consensus has been reached regarding extremity laterality as an ankle sprain risk factor, we tried to investigate if the protective reaction of the peroneal muscles is related to it. The results showed no significant difference between the dominant and non-dominant lower extremities of the participants. Subsequently, this finding implies that in a non-injured athlete, both ankles seem to be under the same protection of the reactive response of the peroneal muscles. Furthermore, since EMD has been suggested to be an indirect sign of muscle stiffness and tone, it can be a useful means to assess joint stability [30]. Thus, the lack of any difference in EMD times might imply that the muscle system in both the dominant and non-dominant leg provides the same ankle joint stability. Most athletes place a greater demand on their dominant limb, and subsequently, they put great effort around the knee and ankle, particularly during high-demand activities that place the ankle and knee at risk [49]. The combination of these assumptions should raise awareness among athletes and consider that their extremities are equally exposed to the danger of an ankle injury.

Another finding of the present study was that both the dominant and non-dominant extremities demonstrated longer EMD after fatigue. The protocol used in this study reinduces fatigue through repetitive isokinetic contractions [36, 50]. Fatigue results in limited force generation capacity during muscle contractions due to the impairment of membrane excitability through various electrolytic disturbances [51, 52]. Fatigue can also affect the neuromuscular mechanism, and as a result, it may induce changes in EMD [34]. Our findings regarding fatigue and EMD agree with previous studies that report an increase in EMD times after fatigue on the peroneal muscles [22] and knee muscles [34, 53]. On the other hand, McLoda et al. [35] did not find any change in peroneal muscle EMD after a task failure fatigue protocol, but they used an induced contraction of the peroneal. Forestier et al., in another study, showed that ankle proprioception is impaired after fatigue [54].

This work might have some important clinical implications since understanding the injury mechanism is an integral part of injury prevention research [55]. The “sequence of injury prevention” has been proposed by van Mechelen et al. and describes how sports injury-related studies came together to form the research framework [56]. Some studies demonstrated decreased peroneal muscle reaction times in healthy subjects after a 6-week neuromuscular training program [57] and a 6-week eccentric/concentric isokinetic training program [40]. These findings imply that the reaction times of the peroneal muscles can be decreased even in healthy subjects. However, it remains questionable whether the improved reflex latencies of the peroneus longus are clinically relevant and could protect an individual from sudden inversion injury.

## Conclusions

In conclusion, we report that laterality does not affect the peroneal longus muscle EMD times. There was no significant difference between dominant and non-dominant ankles in the amateur athlete population. Thus, the protective action of the peroneal muscles is the same at both extremities, and both extremities seem to be equally exposed to an ankle injury. Furthermore, the finding that fatigue causes a significant increase in EMD is concomitant with the current literature. It emphasizes the importance of improving resistance to fatigue in order to prevent delayed peroneal response for both ankles, either dominant leg or not. Combining these findings, training or rehabilitation programs should focus on retraining reaction time to prevent injuries to both legs, either dominant or not. Additionally, isolated and functional fatigue training of the peroneals may add another layer of protection that can potentially prevent ankle sprain recurrence. These may be interesting and potentially useful research questions for future studies aiming to identify and assess intrinsic and extrinsic risk factors of ankle sprains and CAI.
